# Safety and tolerability of adjunct non-invasive vagus nerve stimulation in people with parkinson’s: a study protocol

**DOI:** 10.1186/s12883-023-03081-1

**Published:** 2023-02-03

**Authors:** Hilmar P. Sigurdsson, Heather Hunter, Lisa Alcock, Ross Wilson, Ilse Pienaar, Elizabeth Want, Mark R. Baker, John-Paul Taylor, Lynn Rochester, Alison J. Yarnall

**Affiliations:** 1 Clinical Ageing Research Unit, Campus for Aging and Vitality, Translational and Clinical Research Institute, Faculty of Medical Sciences, Newcastle University, Newcastle Upon Tyne, NE4 5PL Tyne and Wear UK; 2 The Newcastle Upon Tyne Hospitals NHS Foundation Trust, Newcastle Upon Tyne, UK; 3 Institute of Clinical Sciences, University of Birmingham, Edgbaston, Birmingham, B12 2TT UK; 4 Department of Metabolism, Digestion and Reproduction, Imperial College London, London, UK

**Keywords:** Attention, Gait, Cholinergic system, Parkinsons disease, Vagus nerve stimulation

## Abstract

**Background:**

Parkinson’s disease (PD) is the fastest growing neurological condition worldwide. Recent theories suggest that symptoms of PD may arise due to spread of Lewy-body pathology where the process begins in the gut and propagate transynaptically via the vagus nerve to the central nervous system. In PD, gait impairments are common motor manifestations that are progressive and can appear early in the disease course. As therapies to mitigate gait impairments are limited, novel interventions targeting these and their consequences, i.e., reducing the risk of falls, are urgently needed. Non-invasive vagus nerve stimulation (nVNS) is a neuromodulation technique targeting the vagus nerve. We recently showed in a small pilot trial that a single dose of nVNS improved (decreased) discrete gait variability characteristics in those receiving active stimulation relative to those receiving sham stimulation. Further multi-dose, multi-session studies are needed to assess the safety and tolerability of the stimulation and if improvement in gait is sustained over time.

**Design:**

This will be an investigator-initiated, single-site, proof-of-concept, double-blind sham-controlled randomised pilot trial in 40 people with PD. Participants will be randomly assigned on a 1:1 ratio to receive either active or sham transcutaneous cervical VNS. All participants will undergo comprehensive cognitive, autonomic and gait assessments during three sessions over 24 weeks, in addition to remote monitoring of ambulatory activity and falls, and exploratory analyses of cholinergic peripheral plasma markers. The primary outcome measure is the safety and tolerability of multi-dose nVNS in PD. Secondary outcomes include improvements in gait, cognition and autonomic function that will be summarised using descriptive statistics.

**Discussion:**

This study will report on the proportion of eligible and enrolled patients, rates of eligibility and reasons for ineligibility. Adverse events will be recorded informing on the safety and device tolerability in PD. This study will additionally provide us with information for sample size calculations for future studies and evidence whether improvement in gait control is enhanced when nVNS is delivered repeatedly and sustained over time.

**Trial registration:**

This trial is prospectively registered at www.isrctn.com/ISRCTN19394828. Registered August 23, 2021.

## Background

Prevention of immobility and falls in older people is a public health priority. In ageing and age-associated conditions such as Parkinson’s disease (PD), gait disorders and their consequences – most notably falls – are common manifestations. The impact of gait dysfunction and falls in PD is such that people with Parkinson’s (PwP) voted both as the top research priority in a recent James Lind Alliance and Parkinson’s UK priority setting partnership [1].

Gait impairments in PD are evident at the time of diagnosis and respond only selectively to treatment [2]. They have significant consequences, as discrete gait characteristics predict the time to first fall in a cohort of newly diagnosed PwP [3]. Progression of discrete gait impairments such as step time variability and step length variability is evident in early disease despite optimal dopaminergic treatment [4]. This reflects the contemporary view of PD as a complex multisystem disorder in which gait impairments are underpinned by deficits in multiple cortical and subcortical networks as well as age-related neurodegeneration. Novel interventions targeting dopamine-resistant gait impairments and their consequences are urgently needed.

Cognitive impairment contributes to early gait deficits [5] and conversely, gait impairments predict cognitive decline in early PD [6]. There is evidence that the loss of cholinergic neurones from the nucleus basalis of Meynert (nbM) in the basal forebrain and their projections to the frontal cortex is a major contributor to the functional decline in gait, cognition and increased risk of falls [7–10]. For example, we have recently shown that nbM volumes predict future disease-specific gait decline over three years in early PD [10]. In this study, gait pace and variability characteristics were most closely linked with nbM neurodegeneration. Furthermore, a metabolic temporal variability gait covariance network in PD was recently reported to correlate with variability of step and swing time. This network was characterised by predominantly decreased brain glucose metabolism (associated with increased step and swing time variability) in the caudate nucleus, hippocampus, and red nucleus, which all receive cholinergic input from cell groups within the basal forebrain and the pedunculopontine nucleus (PPN) [11]. Research has shown that the number of acetylcholinesterase + neurons in the PPN is reduced in PD fallers relative to those patients without balance deficits and a history of falls [12]. Cholinesterase inhibitors aimed at preventing the breakdown of acetylcholine have demonstrated some promise in the management of PD-associated gait disorder and postural instability [13]. However, drugs are not without side effects and that may be more problematic in older people with multimorbidity.

An alternative approach now being trialled is to implant stimulating electrodes into the nbM [14], although this involves major neurosurgery with equivocal outcomes. A non-invasive technique that may activate the cholinergic circuitry which has gained recent traction in a number of neurological disorders is via non-invasive vagus nerve stimulation (nVNS) [15–17]. The exact underlying mechanisms of action of this method are poorly understood, but an indirect effect impacting the cholinergic system has been postulated [18]. Neuroimaging studies have provided some evidence with increased neural activity observed in multiple regions of the forebrain containing cholinergic neurons including the caudate nucleus and thalamus [19]. Additional corroboration is provided through studies demonstrating attenuation of VNS following the application of the muscarinic agonist scopolamine in a rat model [20] and after lesioning the nbM [15]. More recently, VNS improved locomotion in a rat model of PD [21] and recent randomized controlled trials have additionally demonstrated the feasibility of VNS combined with rehabilitation therapy in improving upper limb motor function in stroke patients [22]. Based on this, adjunctive VNS may be an attractive non-pharmacological approach to improve gait and balance impairments in PD [23].

Research from our group recently assessed the effect of a single dose of nVNS on dopaminergic-resistant gait characteristics (step time variability and step length variability) [4] in a pilot study in 30 PwP [24]. Gait was measured both pre- and post-intervention during a two-minute continuous walk at a comfortable walking pace. All participants were assessed while in an ‘on’ motor state. Participants were randomised to receive either a single dose of active nVNS stimulation or sham stimulation that does not activate the vagus nerve (VN) between the pre- and post-assessments with gammaCore® handheld device, which was applied to the left side of the neck. In the active group, step time variability and step length variability decreased whereas an increase was observed in the sham group, with step length variability reaching statistical significance (-5.6 vs 25.4% change for active vs control group, *p* = 0.045). Decrements in these characteristics represent an improvement; limiting the manifold of gait variability increases gait stability [25].

This exploratory study provides data suggesting that dopamine-resistant gait characteristics improve with nVNS. It is now imperative to ascertain if the effect is enhanced when nVNS is delivered *repeatedly* and *sustained* over time. Therefore, to establish a signal of efficacy and feasibility to deliver in the home, a multi-dose study is now needed.

The aim of the Adjunctive Vagus Nerve Stimulation for Improving Neural Control of Gait in Parkinson’s Disease (AdVaNSING-PD) project is to establish the safety, feasibility, and proof of concept of a non-pharmacological, low-cost, simple-to-use, home-delivered electroceutical approach to boost cholinergic function, improve gait parameters and ultimately reduce falls risk.

## Materials and methods

### Study design

The study is a single-site, proof-of-concept, double-blind sham-controlled randomised pilot trial in patients with PD. Our trial will follow the Standard Protocol Items: Recommendations for Interventional Trials (SPIRIT) guidelines [26, 27]. The study comprises a baseline assessment visit (t_1_) followed by a 12-week treatment phase that includes remote monitoring of ambulatory activity and falls with monthly follow-up calls (t_2_ and t_3_). All participants will be invited for two follow-up visits at 12 weeks (t_4_) and 24 weeks (t_5_) with the latter to assess any long-term retention. At the end of the study, a subset of participants will be invited to contribute to a focus group discussion, to facilitate the shape of a future trial. Table 1 illustrates the SPIRIT flow diagram summarising all study procedures and outcome measures, randomisation, assessments, and interventions for each phase of the study.

**Table 1:** SPIRIT flowchart. Abbrv. TAU, treatment as usual; MoCA, Montreal Cognitive Assessment; MDS-UPDRS-III, Movement Disorder Society-Unified Parkinson’s Disease Rating Scale Part III; H&Y, Hoehn and Yahr staging; PDQ-39, Parkinson’s Disease Questionnaire-39; GDS, Geriatric Depression Scale; CFS, Clinical Frailty Scale; GCSI, Gastroparesis Cardinal Symptom Index; LLFDI, Late-Life Function & Disability Instrument; FACIT-Fatigue, Functional Assessment of Chronic Illness Therapy-Fatigue; SRT, Simple Reaction Time; CRT, Choice Reaction Time; TMT, Trail Making Test; CANTAB, Cambridge Neuropsychological Test Automated Battery; OTS, One Touch Stockings; PAL, Paired Associative Learning

	STUDY PERIOD						
	Enrolment	Allocation	Post-allocation				Close-out
TIMEPOINT	-t_1_	0	t_1_ Start	t_2_ 4weeks	t_3_ 8 weeks	t_4_ 2 weeks	t_5_ 24 weeks
ENROLMENT							
Eligibility screen by care team	X						
Informed consent	X						
Balanced randomisation		X					
Allocation		X					
INTERVENTIONS:							
Active nVNS plus TAU		X					
Sham nVNS plus TAU		X					
ASSESSMENTS							
Questionnaires:							
MoCA			X				
MDS-UPDRS III & H&Y			X			X	X
PDQ-39			X			X	X
GDS-15			X				
CFS (v2.0)			X				
GCSI			X			X	X
LLFDI			X			X	X
FACIT-Fatigue			X			X	X
Device tolerability							X
Neuropsychological tests							
SRT			X			X	X
CRT			X			X	X
Digit vigilance			X			X	X
TMT A & B			X			X	X
CANTAB OTS			X			X	X
CANTAB PAL			X			X	X
Physical assessments							
Hemodynamic & autonomic function			X			X	X
Whole blood sample			X			X	
Grip strength			X			X	X
Gait assessment			X			X	X
Axivity monitoring			X			X	X
Falls			X	X	X	X	X
Adverse events			X	X	X	X	X
Focus group discussion							X

### Study setting

This trial is carried out at the Clinical Ageing Research Unit on the Campus for Ageing and Vitality, Newcastle University, Newcastle upon Tyne.

### Participants and eligibility criteria

We aim to recruit 40 PwP in total. This study will be used to establish proof-of-concept and feasibility allowing power calculation for a larger definitive clinical trial. Participants will be over 18 years of age but younger than 76 years of either sex and of any racial or ethnic origin. The age cut-off will be used due to the lack of safety data in older or younger participants. All participants will have a diagnosis of PD, as per the UK Brain Bank Criteria [28] and participants may be Hoehn and Yahr stages I to III [29]. All participants must be on stable medication for the preceding month and anticipated over the next three months, able to walk independently without aid for a minimum of two minutes without rest and able to provide informed consent. Our exclusion criteria include:

- Parkinson’s disease dementia [30] or significant cognitive impairment as determined by a score of < 21 on the Montreal Cognitive Assessment (MoCA);
- History of stroke, traumatic brain injury, intracranial aneurysm, intracranial haemorrhage, brain tumour or atypical parkinsonian disorder;
- An unstable medical condition in the last six months or planned surgeries that may involve implants within the next six months;
- Prescribed centrally acting anticholinergics (e.g., amitriptyline) or cholinesterase inhibitors;
- Severe orthopaedic or neurological (excluding PD) pathology that will adversely affect gait;
- Pain at the nVNS treatment site (e.g., dysesthesia, neuralgia, cervicalgia);
- Previous use of nVNS stimulator device, including previous participation in nVNS research;
- Women of childbearing potential or who are pregnant or lactating;
- Active participation in another interventional trial or exposure to an experimental drug or intervention;
- Lesion (including lymphadenopathy), previous surgery (including carotid endarterectomy or vascular neck surgery) or abnormal anatomy at the treatment site (open wound, rash, infection, swelling, cut, sore, drug patch, surgical scar[s]);
- Known or suspected severe atherosclerotic cardiovascular disease, severe carotid artery disease (e.g., bruits or history of TIA or stroke), congestive heart failure, known severe coronary artery disease or prior myocardial infarction;
- Abnormal baseline electrocardiogram (ECG) within the last year (e.g., second or third-degree heart block, prolonged QT interval, atrial fibrillation, atrial flutter, history of ventricular tachycardia or ventricular fibrillation);
- Uncontrolled high blood pressure (systolic > 160 mmHg, diastolic > 100 mmHg) after 3 measurements within 24 h;
- Previous unilateral or bilateral vagotomy;
- Implanted metal cervical spine hardware, other metallic implants or implantable medical devices such as deep brain stimulator, hearing aid implant, pacemaker, implanted cardioverter defibrillator, cranial aneurysm and/or cranial aneurysm clips, history of facial/orbital/metallic fragments, implanted electronic device, neurostimulator, valve replacements/stents, metallic implants/prostheses) near the stimulation site such as a bone plate or bone screw;
- History of syncope or seizures (within the last 2 years);
- Patients with active cancer or are in a recent remission of cancer;
- Clinically significant hypotension, bradycardia, or tachycardia;
- Insufficient comprehension of the English language.

The primary objectives are to develop and administer the study protocol, to establish feasibility indicators of the study and to assess compliance, safety, and tolerability of multi-dose nVNS in participants with PD. Key secondary objectives include assessing the impact of multi-dose nVNS treatment on (1) gait as proof of concept. We will be focussing on changes in dopaminergic-resistant gait characteristics including step length variability and step time variability [4] in addition to other discrete (microstructural) characteristics according to an a priori model of gait [31]; (2) changes in macrostructural gait characteristics including volume, variability pattern of walking bouts and steps as measured using seven-day free living monitoring (Axivity monitor); (3) cognition as proof of concept. Particular attention will be paid to changes in cognitive domains relating to attention and fluctuating attention and changes in executive and memory function; and, (4) falls.

### Trial design

The AdVaNSING-PD project is designed as a single-site randomised double-blind sham-controlled feasibility trial with two parallel groups and a primary endpoint of safety and tolerability after 12 weeks of self-administered nVNS with additional follow-up at 24 weeks to assess any carryover effects.

### Randomization and blinding

Patients who provide informed consent to participate and fulfil the eligibility criteria will be equally randomised using a covariate-adaptive randomisation process with a minimisation method [32] into two groups to receive either active nVNS or sham nVNS. This is achieved using the variance minimisation procedure recently proposed [33]. Allocation to groups will be stratified by age, sex, global cognition (MoCA scores) and motor severity (scored using the MDS-UPDRS-III). Both patients and researchers performing the assessments and analyses will be blinded to the intervention.

### Intervention

**Fig. 1:**
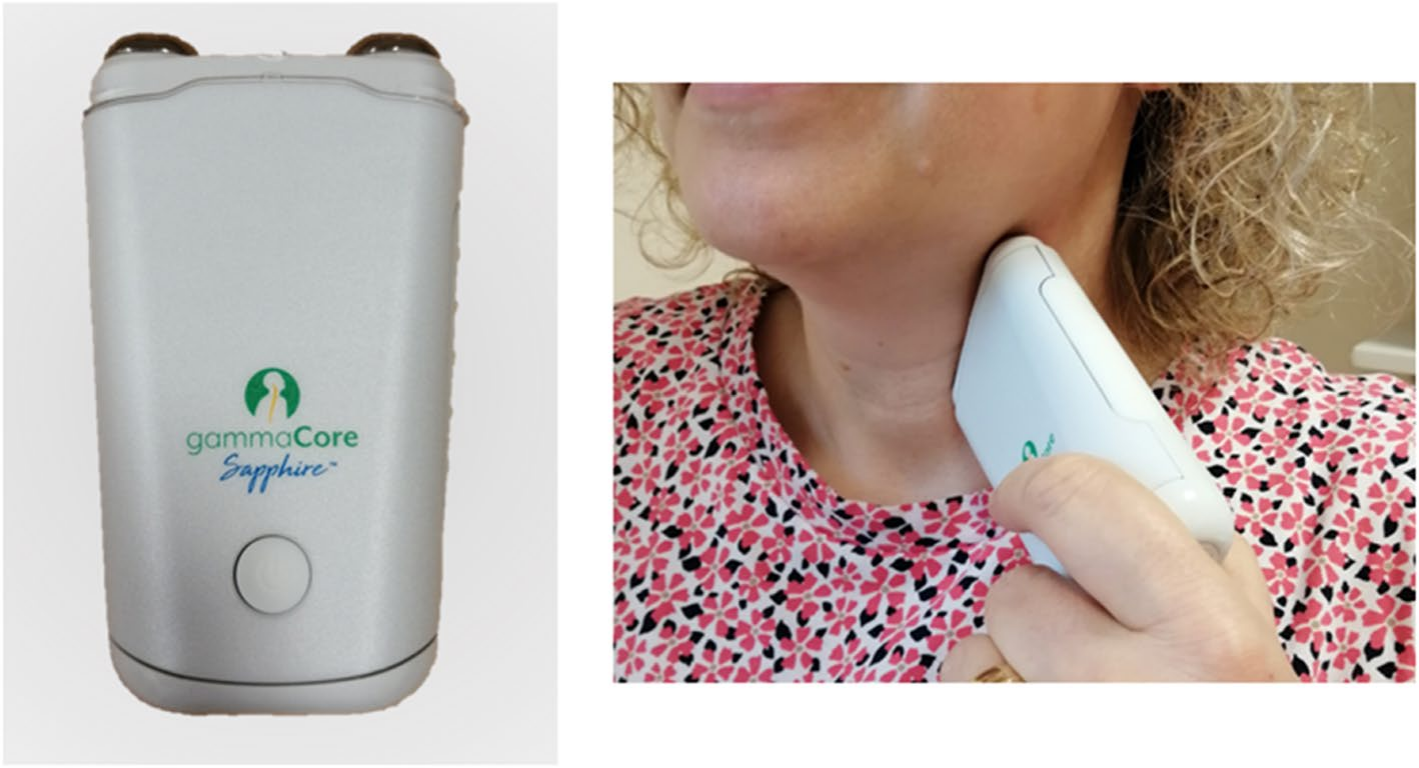
ElectroCore’s gammaCore nVNS device (left) and demonstrated use (right) with the device applied to the left side of the neck

Patients allocated to the *active group* will receive nVNS using the gammaCore® handheld nVNS device (CE 571,753). This is a handheld device that indirectly stimulates the cervical branch of the VN within the carotid sheath. Two flat stimulation contact surfaces emit a low-voltage electrical signal burst which permeates the skin and subcutaneous structures. The electrical signal consists of a 5 kHz sinusoidal wave burst lasting 1 ms that repeats once every 39 ms (25 Hz frequency). The stimulation surface is placed on one side of the neck and the stimulus is applied for 120 s (see Fig. 1). Patients will be instructed to deliver two doses of stimulation 2–5 min apart on the left side, twice daily (in the morning and afternoon) for 12 weeks. The sham devices will be supplied by ElectroCore, LLC, manufacturers of the gammaCore® device. The sham device looks identical to the active nVNS device but does not stimulate the VN. Patients in the *sham group* will receive identical instructions to those in the active group.

Participants in both arms will be shown how to deliver the intervention at the Clinical Ageing Research Unit (Newcastle University) by an unblinded trainer after the participant’s eligibility has been confirmed and randomisation has occurred. The stimulation intensity will be controlled using the device control buttons and ramped up to a comfortable level of intensity. In addition to visual instructions, all participants receive a detailed instruction sheet on how to use the device. Participants will be phoned at weeks 4 and 8 to check progress. Following the 12-week treatment phase, participants will stop using the device, after they have additionally demonstrated their final stimulation to the assessor at the 12-week visit. This is followed by completion of the device tolerability and user questionnaire. Assessors will remain blinded.

The risk associated with the use of the gammaCore® nVNS device is minimal, and the safety profile is strong. The most common adverse events reported include local site tingling, pain, redness or itching and occur in approximately 17% of participants [34]. These adverse events are quickly resolved following discontinuation of the use of the device. Due to the theoretical risk of increased cardiac adverse events with stimulation of the right VN, participants will be asked to stimulate the left side [35]. Any adverse events (AE) will be diarised during the intervention and closely monitored until resolution, stabilisation or until it has been shown that the study procedures are not the cause. Participants who discontinue or deviate from the intervention protocol will be retained in the study as intention to treat.

### Sample size

The purpose of this study is to establish proof of concept and feasibility for a larger clinical trial and the number of participants recruited for this study (*n* = 40) matches that of a recent feasibility trial [36]. This study will therefore establish a proof of concept and allow power calculations for a definitive trial.

### Assessment procedures

All patients will be assessed while in an ‘on’ motor state, taking their medication as prescribed. Participants will undergo a baseline visit to assess study eligibility by a clinician (AJY). This will include a detailed clinical review to encompass demographic and anthropometric data, falls history and medication. This clinical review will additionally include haemodynamic and autonomic function assessment including a 12-lead electrocardiogram (ECG) recorded using a bedside monitor. The ECG will be scrutinised by a clinician (AJY) for cardiac abnormalities. Haemodynamic parameters including heart rate, systolic and diastolic blood pressure will be recorded. In addition, five minutes of artefact-free R-R (beat-to-beat) interval data will be recorded. Quantitative markers of autonomic activity – both in time and frequency domains – will be computed according to the Task Force of the European Society of Cardiology [37], including heart rate variability (HRV).

Health-related quality of life, depression, global cognition and motor evaluation will be assessed using the Parkinson’s Disease Questionnaire (PDQ-39) [38], Geriatric Depression Scale-15 (GDS-15) [39], the Montreal Cognitive Assessment (MoCA) [40] and the MDS Unified Parkinson’s Disease Rating Scale III (MDS-UPDRS III) [41] plus Hoehn and Yahr staging [29], respectively. Hand grip strength will be measured with a Jamar handheld hydraulic dynamometer (Promedics, UK) acquiring three trials bilaterally following a standard protocol [42]. Furthermore, we will employ the Clinical Frailty Scale (CFS) [43] to estimate frailty status in the two weeks before assessment; the Gastroparesis Cardinal Symptom Index (GCSI) [44] to explore the impact of nVNS on gastroparesis symptoms; the Late-Life Function & Disability Instrument (LLFDI) to measure deficits in function and disability [45]; and the Functional Assessment of Chronic Illness Therapy-Fatigue Scale (FACIT-Fatigue) [46–48] to monitor fatigue.

### Primary outcome measures

We will primarily be examining the safety of the intervention in PwP and device tolerability by recording all adverse events reported by patients. Also, the proportion of eligible patients who agree to participate in the trial and the proportion of enrolled patients who successfully complete follow-ups will be monitored. During study visits, we will ask the participants to report any issues with using the device.

### Secondary outcome measures

#### Gait

Gait and posture will be assessed pre- and post-intervention during a two-minute continuous walk using an instrumented walkway and a small, lightweight, sensor worn on the lower back (AX6, Axivity, York, UK; sampling frequency = 100 Hz, gravitational acceleration =  ± 8 g, and gyroscope full-scale range (dps) = 2000, attached using double sided tape and secured in place using hypafix [BNS Medical Limited, Hull, UK]). The sensor is an inertial measurement unit comprising a tri-axial accelerometer and gyroscope. Participants will walk at a comfortable walking pace around a 25 m circuit inclusive of a 6.1 × 0.6 m instrumented walkway (ProtoKinetics LLC, Havertown, Pennsylvania, 120 Hz). The analysis will focus on dopa-resistant characteristics; namely, step time variability and step length variability [4, 13, 49], and other discrete characteristics representing five domains according to an a priori model of gait in PwP (see Table 2 for details) [50].

**Table 2:** Description of the validated model of gait in PwP as described by Lord and colleagues [50]

	Gait domain	Description
Validated model of gait in PwP	Pace	The pace domain is captured by characteristics such as step velocity and step length
	Rhythm	The rhythm domain presents the temporal regulation of gait and the timing of steps such as step time, swing time and stance time
	Variability	The variability domain represents the differences from one step to the next. This domain contains variability gait characteristics such as the variability of step length, step time and stance time
	Asymmetry	The asymmetry domain represents the temporal differences between left and right steps and includes gait characteristics measuring the asymmetry of swing time, step time and stance time
	Postural control	The postural control domain represents the ability to maintain balance during walking with gait characteristics including mean step width

### Ambulatory activity

Ambulatory activity will be measured in the real-world using a body-worn sensor on the lower back (same sensor as used in the laboratory assessment, attached using hydrogel patch [Amgel Technologies, USA] and secured in place using hypafix). Participants will be asked to wear the sensor for seven consecutive days to provide free-living data in terms of gait activity. Outcome measures will be in accordance with published work [51, 52]. Description of these measures, which can be broadly categorised into three domains including volume, pattern, and variability (macro gait characteristics), is described briefly in Table 3.

**Table 3:** Description of macro (behavioural) gait characteristics quantified from wearable sensors worn for seven days

	Category	Description
Macro (behavioural) gait characteristics	Volume	Volumetric outcomes describe the quantity or amount of activity levels. From this total walking time, steps per day and bouts per day will be reported
	Pattern	Pattern outcomes describe the type of walking behaviour and will include descriptors such as mean bout length (based on ambulatory bouts) and the non-linear descriptor *alpha*. This variable captures the distribution of ambulatory bouts and is the ratio of short to long bouts
	Variability	The variability outcome describes the within-subject variation of bout length capturing walking activity. Higher variability is interpreted to indicate more varied walking patterns whereas lower variability indicates more repetitive and habitual levels of activity

Previous work from our group indicates the sensor is well tolerated [51, 53, 54]. Macro gait characteristics will be measured following baseline (t_1_), the 12-week treatment phase (t_4_) and the 24-week assessment (t_5_).

### Cognition

**Fig. 2:**
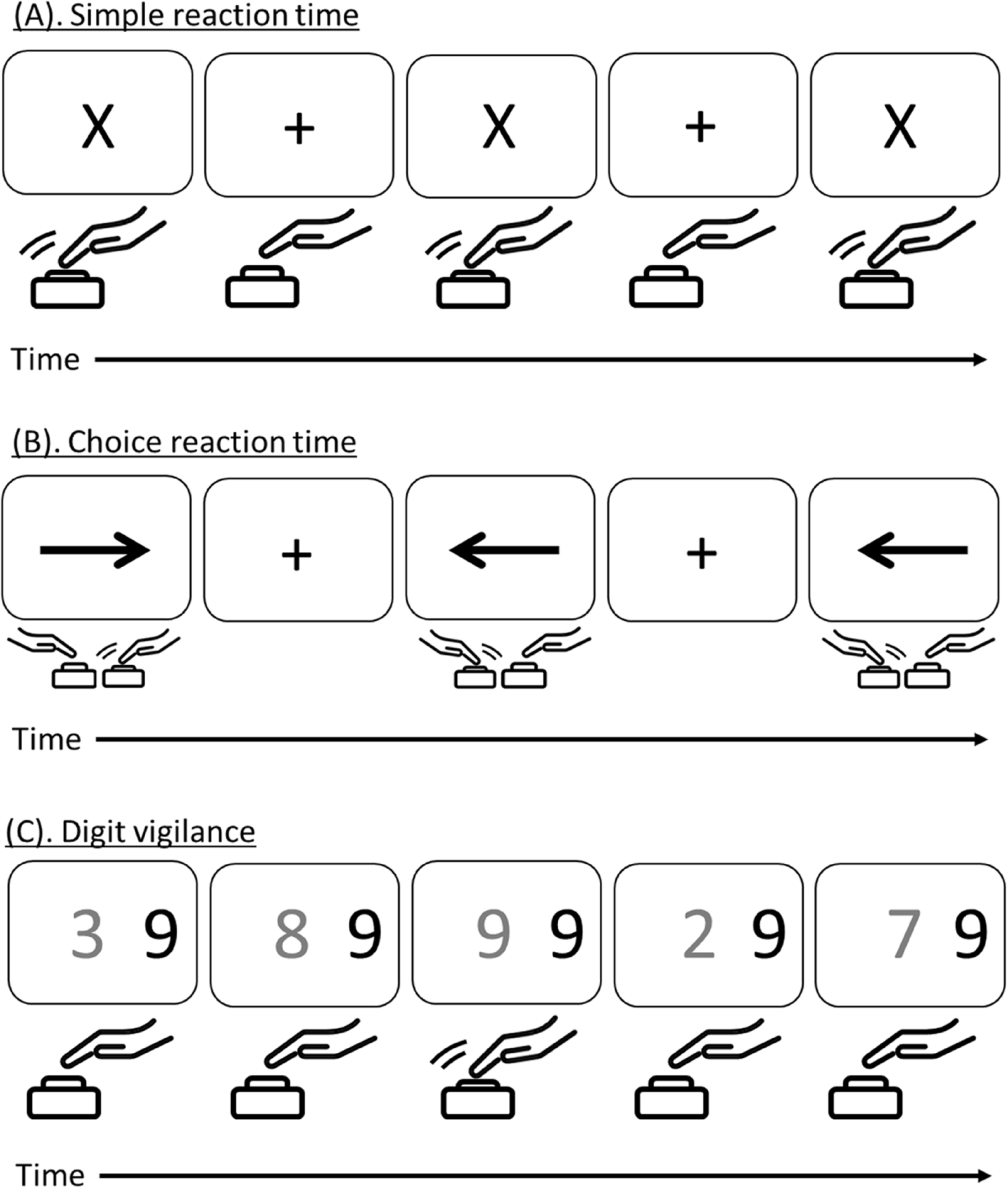
The figure illustrates the three cognitive assessments: simple reaction time task (**A**), choice reaction time task (**B**) and Digit vigilance task (**C**) collectively measuring attention and fluctuating attention. Hand icon represents the participant’s hand responding to the stimuli displayed on the computer screen. For SRT and DV the dominant hand is used

Attention and fluctuating attention will be measured using a computerised battery by assessing mean reaction time during simple reaction time (SRT), choice reaction time (CRT) and digit vigilance (DV) tests (examples of these tests are depicted in Fig. 2) [55, 56]. Power of attention (PoA) will be computed as a marker of attention and is the composite score of mean reaction time from all three tests reported in milliseconds. Fluctuating attention will be determined by the coefficient of variance (CoV) of the three tests. Also, digit vigilance accuracy will be reported as the percentage of correct targets detected.

Executive and memory functions will be evaluated using the computerised Cambridge Neuropsychological Test Automated Battery (CANTAB) [57]. Executive function will be assessed using the One Touch Stockings of Cambridge (OTS) and Paired Associates Learning (PAL) will be used to assess memory. The use of these two assessments is informed by previous research [56, 58]. The Trail Making Test (TMT) parts A and B will be used to assess attention and executive function, respectively.

### Falls

Consistent with the recommendations of the Prevention of Falls Network Europe group falls will be defined as “an unexpected event in which the participant comes to rest on the ground, floor, or lower level” [59]. These will be recorded prospectively using standardised falls diaries, sent out on a monthly basis with a pre-paid envelope and regular telephone calls from a study team member, to verify the information [60, 61].

### Cholinergic peripheral plasma markers

Approximately 14 ml of whole blood will be taken from participants pre- and post-intervention. Samples will be used to measure cholinergic peripheral markers in plasma. Metabolomic analysis of blood plasma concentrations of protein elements of the cholinergic metabolic pathway will be completed by external collaborators. Plasma will be analysed, and protein elements involved in the cholinergic (Cho) metabolic pathway will be identified and quantified. Assays will be validated for specificity, linearity, accuracy, precision, recovery, and stability according to FDA bio-analytical method validation guidelines [62].

### Focus group

The primary goal of our focus group, which will be held with a subset of participants towards the end of the trial, will be to co-plan and co-design a future, multi-site nVNS trial in PwP.

### Statistical analysis

The main analyses will be descriptive, to inform the design of a future trial. We will analyse the numbers of eligible patients enrolled, rate of recruitment, compliance with the intervention and completion of study assessments. We will ascertain the completeness and acceptability of nVNS and identify any potential barriers. Qualitative data analysis from the focus group will adopt a thematic approach [63] with NVivo (QSR International Pty Ltd. NVivo) software used as a coding tool. Statistical analyses will be conducted using MATLAB (The MathWorks, Inc., Natick, MA, USA), SPSS (IBM SPSS Statistics for Windows, Armonk, NY: IBM Corp) and/or R (http://www.r-project.org/). The outcome data will be summarised using descriptive statistics, including means (and medians), standard deviations, and interquartile ranges. It is expected that non-parametric statistics (e.g., Friedman test) will be used throughout due to the small number of participants in this study. To establish proof of concept for active nVNS treatment, we will compare the median change scores (post–pre assessment) of gait and cognitive outcomes between groups using Mann–Whitney U tests. An exploratory outcome will be a metabolomic analysis of cholinergic markers in blood plasma. We will explore the effects of concentrations of the various targeted small molecule Cho metabolites (Cho inclusive) in plasma on cognitive functions and discrete gait characteristics.

## Discussion

nVNS is a non-pharmacological neuromodulation intervention currently approved by the FDA for the acute and preventive treatment of most forms of primary headache and received a CE mark from the Medicines and Healthcare products Regulatory Agency (MHRA) for the treatment of primary headache, as adjunctive therapy to reduce symptoms of certain anxiety and depression conditions and to reduce symptoms of gastric motility disorders and irritable bowel syndrome. nVNS furthermore has some evidence in improving symptoms in conditions including fatigue and systemic inflammation [64] which are characteristic features of PD.

In PD, previous investigations have shown that single-dose nVNS [24, 65] may improve discrete gait characteristics. However, no formal investigation of the safety and tolerability of the device has been conducted, especially in a multi-dose nVNS trial. According to findings from a large meta-analysis that synthesised all reported adverse events from studies, the safety profile of vagal stimulation is excellent [34].

In this 24-week feasibility trial, we aim to generate clinical, digital and laboratory data to assess the safety, tolerability, feasibility, and potential effectiveness of domiciliary multi-dose nVNS in people with Parkinson’s. Further outcomes from this study include metrics needed for sample size calculations for future studies and evidence that improvement in gait, cognition and autonomic function is enhanced when nVNS is delivered repeatedly and whether this is sustained over time. Critically, these study outcomes will be used to provide input to a future larger multi-centre trial with benefits seen for patients within the next few years.

## Data Availability

Not applicable. Data will be pseudo-anonymised using a unique identification code assigned at the first assessment and entered to a secure data system on Newcastle University computers for analysis.

## Abbreviations

CANTAB: Cambridge neuropsychological test automated battery
CFS: Clinical frailty scale
CRT: Choice reaction time
DV: Digit vigilance
ECG: Electrocardiography
FACIT-Fatigue: Functional assessment of chronic illness therapy-fatigue
GCSI: Gastroparesis cardinal symptom index
GDS: Geriatric depression scale
HRV: Heart rate variability
LLFDI: Late-life function & disability instrument
MDS-UPDRS: Movement disorders society-unified parkinson's disease rating scale
MHRA: Medicines and healthcare products regulatory agency
nbM: Nucleus basalis of meynert
OTS: One touch stockings
PAL: Paired associative learning
PD: Parkinson's disease
PDQ-39: Parkinson's disease questionnaire-39
PwP: People with parkinson‘s
REC: Research ethics committee
SRT: Simple reaction time
TAU: Treatment as usual
TMT: Trail making test
VN: Vagus nerve
VNS: Vagus nerve stimulation
